# Plumbagin Improves Cognitive Function via Attenuating Hippocampal Inflammation in Valproic Acid-Induced Autism Model

**DOI:** 10.3390/brainsci15080798

**Published:** 2025-07-27

**Authors:** Nasrin Nosratiyan, Maryam Ghasemi-Kasman, Mohsen Pourghasem, Farideh Feizi, Farzin Sadeghi

**Affiliations:** 1 Student Research Committee, Babol University of Medical Sciences, Babol P.O. Box 4136747176, Iran; nosrati.nasi68@gmail.com; 2 Department of Anatomy, Embryology and Histology, Faculty of Medicine, Babol University of Medical Sciences, Babol P.O. Box 4136747176, Iran; faridehfeizi@yahoo.com; 3 Cellular and Molecular Biology Research Center, Health Research Institute, Babol University of Medical Sciences, Babol P.O. Box 4136747176, Iran; sadeghifarzin6@gmail.com; 4 Department of Physiology, School of Medicine, Babol University of Medical Sciences, Babol P.O. Box 4136747176, Iran; 5 Department of Medical Virology and Biotechnology, Faculty of Medicine, Babol University of Medical Sciences, Babol P.O. Box 4136747176, Iran

**Keywords:** neuroinflammation, autism, plumbagin, valproic acid, hippocampus

## Abstract

**Background/Objectives:** The hippocampus is an essential part of the central nervous system (CNS); it plays a significant role in social–cognitive memory processing. Prenatal exposure to valproic acid (VPA) can lead to impaired hippocampal functions. In this study, we evaluated the effect of plumbagin (PLB) as a natural product on spatial learning and memory, neuro-morphological changes, and inflammation levels in a VPA-induced autism model during adolescence. **Methods:** Pregnant Wistar rats received a single intraperitoneal (i.p.) injection of VPA (600 mg/kg) or saline on gestational day 12.5. The male offspring were then categorized and assigned to five groups: Saline+DMSO-, VPA+DMSO-, and VPA+PLB-treated groups at doses of 0.25, 0.5, or 1 mg/kg. Spatial learning and memory were evaluated using the Morris water maze. Histopathological evaluations of the hippocampus were performed using Nissl and hematoxylin–eosin staining, as well as immunofluorescence. The pro-inflammatory cytokine levels were also quantified by quantitative real-time PCR. **Results:** The findings revealed that a VPA injection on gestational day 12.5 is associated with cognitive impairments in male pups, including a longer escape latency and traveled distance, as well as decreased time spent in the target quadrant. Treatment with PLB significantly enhanced the cognitive function, reduced dark cells, and ameliorated neuronal–morphological alterations in the hippocampus of VPA-exposed rats. Moreover, PLB was found to reduce astrocyte activation and the expression levels of pro-inflammatory cytokines. **Conclusions:** These findings suggest that PLB partly mitigates VPA-induced cognitive deficits by ameliorating hippocampal inflammation levels.

## 1. Introduction

Autism spectrum disorder (ASD) is a complex neurodevelopmental disorder characterized by two core symptoms: social impairments and repetitive behaviors [1]. Additionally, deficits in non-social cognitive functions, particularly in hippocampal-related spatial learning and memory, are commonly observed in individuals with autism. Notably, ASD symptoms manifest within the first two years of life, a critical period for hippocampal development [2]. Therefore, highlighting the hippocampus as a prime brain region implicated in ASD could represent a promising approach to its treatment.

Recent evidence suggests that inflammatory mechanisms may be a key factor in understanding the etiology and pathogenesis of ASD [3]. Clinical studies have reported elevated levels of pro-inflammatory cytokines such as IL-6, TNF-α, and IL-1β in the plasma, blood mononuclear cells, serum, and cerebrospinal fluid (CSF) of autistic patients [4,5]. Studies have also shown neuropathological changes in both human brain samples and animal models of autism [6,7]. Based on this evidence, neuroinflammation can be considered a diagnostic indicator and a therapeutic target for ASD. These observations emphasize the contribution of an impaired immune system to the neuropathology of autism.

Valproic acid (VPA) (2-propylpentanoic acid) is a common drug used to treat seizures, stabilize mood, and manage migraines [8]. Among the genetic and non-genetic (environment) risk factors for ASD, VPA exposure during the neurulation period (e.g., gestational day 12.5 in rodents) is considered a well-established environmental model [9].This model reproduces behavioral abnormalities and molecular/cellular mechanisms associated with ASD [10].

Nowadays, natural naphthoquinones are widely utilized in drug design approaches [11]. The low-molecular-weight vitamin K3 analog plumbagin (PLB) (5-hydroxy-2-methyl-1,4-naphthoquinone), derived from *Plumbago zeylanica* roots, readily crosses the blood–brain barrier (BBB) [12,13]. PLB, a natural quinone with a multi-target profile, exhibits strong biological properties. At low doses, it demonstrates robust antioxidant and anti-inflammatory activities by mediating Nrf2 activation and downregulating the NF-κB signaling pathway [14]. Recent studies have highlighted PLB’s potential neuroprotective properties, suggesting its efficacy as a pharmacological treatment for neurodegenerative and neuroinflammatory disorders, including Parkinson’s and Alzheimer’s diseases [15,16]. Moreover, low-dose administration has been proven to ameliorate anxiety-like behavior, amnesia, depression-like symptoms, and memory deficits because of its powerful antioxidant and anti-inflammatory mechanisms [17,18,19]. Our recent study demonstrated that PLB mitigated social behavior impairments in the VPA model of autism by reducing oxidative stress and inflammatory responses in the cerebellum [20].

Considering the important roles of the hippocampus in encoding spatial learning and memory, the present study aims to investigate the impact of PLB administration on the cognitive function and hippocampal inflammation levels in a VPA-induced autism model.

## 2. Materials and Methods

### 2.1. Chemicals

VPA, PLB, and dimethylsulfoxide (DMSO) were purchased from Sigma-Aldrich (St. Louis, MO, USA). Chloral hydrate was obtained from Merck (Germany).

### 2.2. Animals

From the animal house at the University of Medical Sciences in Babol (Iran), we received twelve female and six male adult albino Wistar rats (between 200 and 250 g). The animals were kept under controlled laboratory conditions of a temperature of 22 ± 2 °C and a 12 h light/dark cycle. The rats were given free access to food and water. All the experimental procedures were approved by the ethics committee of the Babol University of Medical Sciences (approval No: IR.MUBABOL.HRI.REC.1398.058).

### 2.3. Induction of Experimental Model of Autism

Based on the previous study, the reproductive cycle for each female rat was determined by the vaginal smear method and the rats were mated overnight (2:1). A confirmation of pregnancy was obtained by observing the vaginal plaque or sperm in the vaginal smear [21].

On day 12.5 of gestation (during fetal neural tube development), ten pregnant rats were given VPA dissolved in saline (600 mg/kg) via an intraperitoneal (i.p.) injection, while two pregnant rats were given only saline. The VPA dosage volume was 0.25 mL per rat. Three days after the birth of the pups, only the male rats were randomly assigned to 5 groups. The number of animals was 6 rats in each group (n = 6).

### 2.4. Experimental Groups

The litter pups were kept with the mothers until the end of lactation (postnatal day (PND) 21) and treated on PND 7 to PND 35 (adolescent) according to the following groups:

Group I. Saline+DMSO: The pups whose mothers received saline were given DMSO 0.1% orally on PND 7.

Group II. VPA+DMSO: The pups whose mothers received VPA on gestation day 12.5 were given 0.1% DMSO orally on PND 7.

Group IIIV. VPA+PLB: The pups whose mothers received VPA were given oral gavage of PLB at doses of 0.25, 0.5, or 1 mg/kg on PND 7, with a daily administration volume of 0.05 mL per rat.

### 2.5. Morris Water Maze Test

The MWM test [16] was used to evaluate the spatial learning and memory in pups between postnatal days 30 and 34. The maze was a dark, circular tank (diameter of 150 cm, depth of 50 cm) equally divided into four quarters (S, N, E, and W). The depth of the water in the maze was 30 cm at room temperature (22 ± 1 °C). A hidden stage (10 cm diameter) was kept at a 2 cm depth from the water surface, known as the SE quadrant. Four black-and-white signs mounted on the wall were visible to the pups. There was also a camera above the tank that recorded the path of the pups. This computerized tracking was recorded by the HVS Image application (UK), which collected data for the final analysis.

This task was performed over 5 days and consisted of two phases, place navigation and the probe trial. In place navigation, the pups were subjected to four trials per day. During place navigation, the habituation trials were performed. The pups were released into the water by the tail and had a 60 s opportunity to find the hidden stage. If the pup found the stage within 60 s, the trial would be stopped and the pup was permitted to remain on the stage for 10 s. If the pup failed to locate the stage, it was gently guided to the stage and allowed to remain there for 20 s. In other trials, each pup was placed into the water from a different quadrant and allowed to search for the hidden stage. The parameters of escape latency, speed, and distance moved were determined every day for each pup and an analysis of the data was performed using the EthoVision XT video tracking software (Noldus Information Technology, Germany). On the fifth day, during the probe trial, the hidden stage was moved out of the tank, and the pups were released from the N quadrant. Each pup was allowed 60 s to swim freely in the tank, during which time the duration spent in the target quadrant was recorded.

### 2.6. Histological Staining

After performing the behavioral tests on postnatal day (PND) 35, the pups were anesthetized with chloral hydrate at a dose of 300 mg/kg, and brain samples were carefully removed from their skulls. The left hemispheres of the brains were incubated in a 4% paraformaldehyde (PFA) solution. After 16 h, for the immunofluorescence analysis, three hemispheres from each group were cryoprotected by storing them in a 30% sucrose solution maintained at 4 °C before being sectioned. Three hemispheres from each group were fixed in another fresh 4% paraformaldehyde (PFA) solution for Nissl and H&E staining. After two weeks, the brain samples were embedded in paraffin. To minimize an anatomical continuity overlap, serial sections were collected at intervals of 60–70 µm.

#### 2.6.1. Nissl Staining

Neuronal cell damage in the hippocampus regions (CA1, CA2, CA3, and DG) was assessed using Nissl staining. Sagittal sections (6 μm thick) were prepared using a Leitz 1512 microtome (Germany), based on our previous report [22]. In brief, the tissue was mounted on slides and then deparaffinized and dehydrated using xylene and alcohol. For five minutes, the slides were incubated in a cresyl violet solution (Merck, Germany) at 37 °C. The sections were dehydrated again using alcohol and cover-slipped. The hippocampus was evaluated and photographed using an Olympus IX71 microscope (Japan). For the histological analysis, we chose three slides from each hippocampus, and three hippocampi from each group (overall, 8–9 sections from each group).

#### 2.6.2. Hematoxylin and Eosin (H&E) Staining

Neuro-morphological alterations were assessed using H&E staining in the hippocampus. H&E staining was performed based on our previous study [23]. The photographs of the hippocampus regions were taken with an Olympus IX71 microscope (Japan). In line with previous studies, morphological changes, including the presence of pyknotic cells with darkly stained nuclei, vacuolation indicative of cell loss, and a disorganized cellular arrangement, were assessed and subsequently scored in a blinded manner by three independent persons [24,25]. To evaluate the differences among groups, the count of pyknotic cells with dark nuclei staining was categorized into four degrees, as shown in Table 1. The results were arranged in four degrees as follows: none (0), moderate (1), mild (2), and severe (3), respectively. For the histological analysis, we chose four slides from each pup, and three pups were used from each group (overall, twelve sections for each group). Consensus was achieved through the use of clearly defined morphological criteria, observer training on representative hippocampal sections, and independent blinded scoring. Qualitative observations were systematically converted into semi-quantitative scores using a standardized grading framework.

### 2.7. Immunohistofluorescence

Astrocyte marker (glial fibrillary acetic acid protein (GFAP)) expression was assessed by immunohistofluorescence staining, as we mentioned previously [26]. In brief, the hippocampus sections were rinsed using PBS and permeabilized with normal goat serum (NGS 10%) in 0.3% Triton X100 for 60 min to block non-specific bindings. Rabbit anti-GFAP (Z0334, Dako) was applied at a 1:400 dilution and the sections were incubated overnight at 4 °C. Following washes in PBS, the hippocampal sections were incubated with a secondary antibody (anti-rabbit immunoglobulin G (IgG) conjugated with Alexa 594 (1:1000, Abcam, UK)) for 60 min at room temperature. Following this, the hippocampal sections underwent three PBS washes before DAPI (4′,6-diamidino-2-phenylindole). The slides were evaluated using a fluorescent microscope (Olympus-microscope IX71, Japan). We used the Image J software (NIH) to analyze the immunostaining data. Three slides were obtained from each pup, and three pups were used from each group for histological data quantification (overall, nine for each group).

### 2.8. Quantitative Real-Time Polymerase Chain Reaction (qRT-PCR)

At PND 35, the animals received a 300 mg/kg i.p. injection of chloral hydrate for anesthesia before sacrifice. The hippocampus was collected from the right hemisphere. The samples were stored in an RNAlater solution overnight before being kept at −80 °C. A Total RNA Extraction Kit from Parstous Company (Iran) was used for RNA isolation. The RNA concentration and quality were determined using a Nanodrop Spectrophotometer (ThemoFisher Scientific) and gel electrophoresis. After that, cDNA was synthesized (Parstous Company, Iran). The qRT-PCR was performed on a QIAGEN (Germany) system with Ampliqon (Denmark) 2x SYBR Green master mix. In brief, the reaction (20 μL) contained SYBR Green master mix (10 μL), the forward primer and reverse primer (0.5 μL each) (Takapouzist, Iran), DEPC-treated water (6 μL), and cDNA (3 μL). Table 2 lists the primer sequences for *IL-6*, *TNF-α*, *IL-1B*, and *GAPDH*. The cycle thresholds were calculated using the LinRegPCR software (version 7.1). A gene expression analysis was performed by using the “Pfaffl” method. The log2-fold-change was calculated to normalize the data, and *GAPDH* was used as a housekeeping gene.

### 2.9. Statistical Analysis

The data were analyzed with the GraphPad Prism version 9 software (La Jolla, CA, USA). The normality of the data was assessed using the Kolmogorov–Smirnov and Shapiro–Wilk tests. For the MWM test, repeated-measures ANOVA tests were performed. The histological data, immunostaining, the probe test, and real-time PCR were statistically analyzed using a one-way ANOVA and Tukey’s post hoc test. The mean (±SEM) of the data is presented. Statistical significance was defined as a *p* < 0.05.

## 3. Results

### 3.1. Plumbagin Improved Spatial Learning and Memory in the VPA-Induced Autism Model

**Figure 1:**
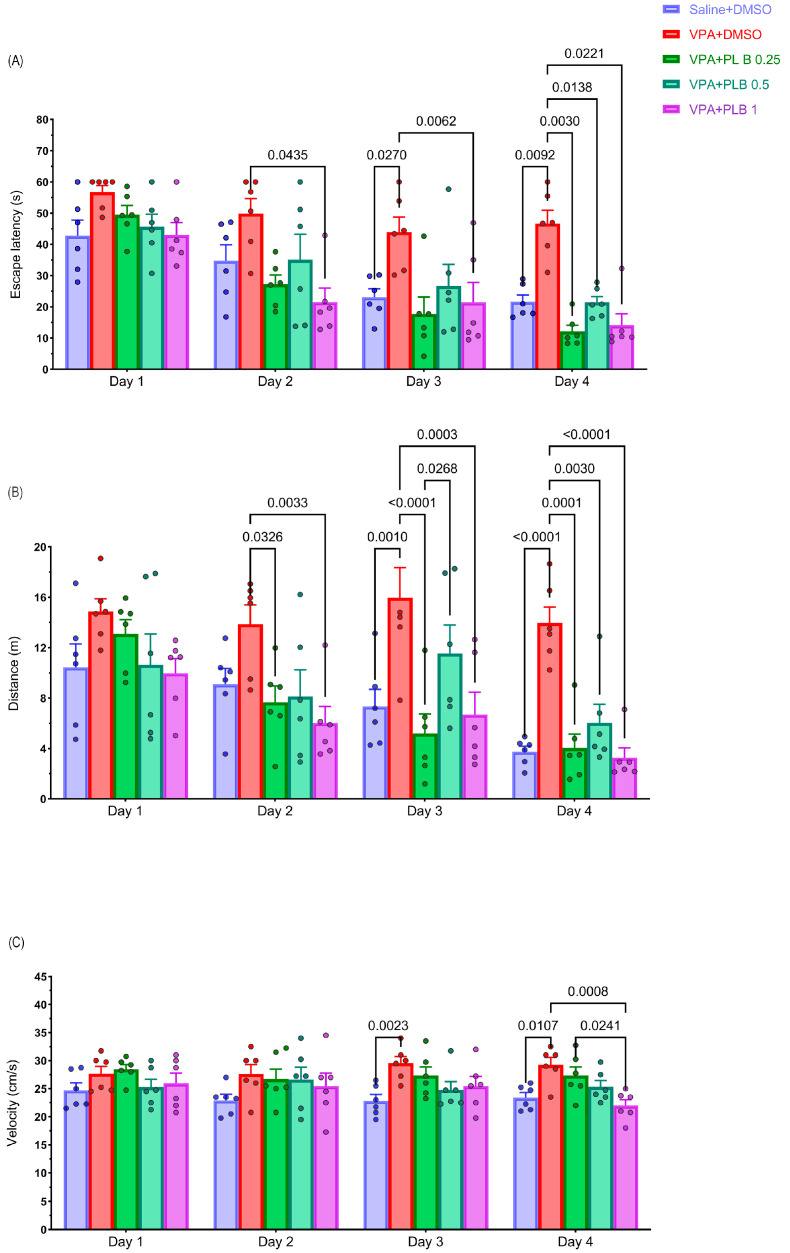
Effect of PLB on spatial learning of rats in VPA-induced autism model. Representative MWM test on PNDs 30 to 34. (**A**) Escape latency, (**B**) traveled distance, and (**C**) velocity were assessed for four days. Data represented as mean ± SEM (n = 6).

During the MWM test conducted on PNDs 30 to 33, we assessed the escape latency, traveled distance, and velocity. The repeated-measures test revealed a significant difference in the escape latency time (F (2.474, 12.37) = 38.57, *p* < 0.0001), as well as in the groups (F (1.9, 9.5) = 12.93, *p* = 0.002). The interaction did not reach statistical significance (F (3.42, 17.10) = 1.129, *p* = 0.37). Our data revealed that pups in the VPA+DMSO-treated group exhibited a longer latency in finding the platform, particularly on the third day (*p* = 0.02) and fourth day (*p* = 0.009), compared to the saline+DMSO-treated pups, indicating a disability in spatial learning. Notably, treatment with PLB resulted in a significant reduction in latency for finding the platform on day 3 for the VPA+PLB 1-treated pups (*p* = 0.006) and on day 4 for the VPA+PLB 0.25-treated pups (*p* = 0.003), VPA+PLB 0.5-treated pups (*p* = 0.01), and VPA+PLB 1-treated pups (*p* = 0.02) when compared to the VPA+DMSO-treated pups (Figure 1A).

The repeated-measures analysis of the traveled distance revealed a significant difference in the time (F (3, 15) = 11.38, *p* = 0.0004) among groups (F (4, 20) = 16.29, *p* < 0.0001). However, their interaction did not reach statistical significance (F (12, 60) = 1.52, *p* = 0.1002). On the second day, the distance traveled decreased in the VPA+PLB 0.25-treated pups (*p* = 0.04) and VPA+PLB 1-treated pups (*p* = 0.004) compared to the VPA+DMSO-treated pups. Furthermore, on both the third and fourth days, the distance traveled by the VPA+DMSO-treated pups was significantly longer than the saline+DMSO-treated pups on day 3 (*p* = 0.0011) and day 4 (*p* < 0.0001), emphasizing a disability in spatial learning. All the groups treated with PLB exhibited a substantial decrease in the traveled distance compared to the VPA+DMSO-treated pups. On day 3, the VPA+PLB 0.25-treated pups (*p* < 0.0001) and VPA+PLB 1-treated pups (*p* = 0.0004) showed a significant decrease in the traveled distance. On day 4, the VPA+PLB 0.25-treated pups (*p* = 0.001) and VPA+PLB 1-treated pups (*p* < 0.0001) exhibited a significant decrease in the traveled distance compared to the VPA+DMSO pups. Additionally, the VPA+PLB 0.5-treated pups showed a significant decrease on day 4 (*p* = 0.003) compared to the VPA+DMSO pups (Figure 1B).

A repeated-measures test also indicated that the velocity was not significant at times (F (3, 15) = 0.618, *p* = 0.6136). In contrast, there was a significant effect for the groups (F (4, 20) = 3.122, *p* = 0.0379). Additionally, the interaction did not reach statistical significance (F (12, 60) = 0.925, *p* = 0.527). However, the velocity analysis revealed a significant increase in the velocity of the VPA+DMSO-treated pups on day 3 (*p* = 0.002) and day 4 (*p* = 0.01) compared to the saline+DMSO-treated pups. Additionally, on the fourth day, the velocity was significantly reduced for the VPA+PLB 1-treated pups (*p* = 0.0007) compared with the VPA+DMSO-treated pups and the VPA+PLB 0.25 pups (Figure 1C).

**Figure 2:**
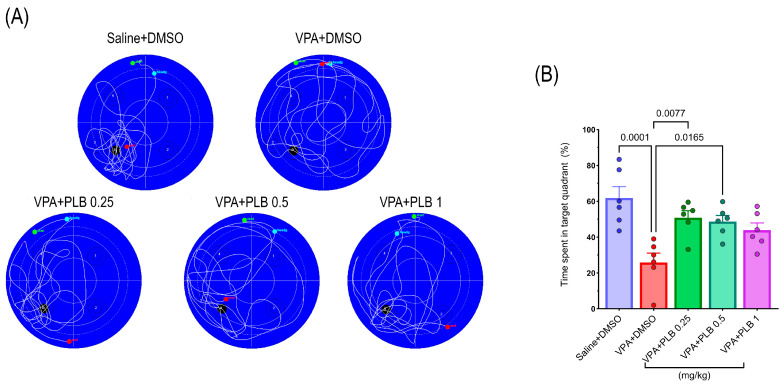
Effect of PLB on spatial memory of rats in VPA-induced autism model. (**A**) Swim track illustrations of prob test for all groups on day 34. The black circle refers to previous hidden platform. (**B**) Analysis of probe test data. Data represented as mean ± SEM (n = 6).

At PND 34, the spatial memory was tested with a single probe trial. The one-way ANOVA showed a significant effect between groups (F (4, 25) = 7.74, *p* = 0.0003). Based on the post hoc analyses, the percentage of time spent in the target quadrant significantly decreased in the VPA+DMSO pups (*p* = 0.0001) compared with the saline+DMSO-treated pups. Conversely, the groups treated with PLB 0.25 (*p* = 0.007) or 0.5 mg/kg (*p* = 0.01) exhibited a significant increase in the time spent in the target quadrant compared with the VPA+DMSO group, suggesting an enhanced spatial memory (Figure 2A,B).

### 3.2. Plumbagin Reduced the Number of Hippocampal Dark Cells in the VPA-Induced Autism Model

**Figure 3:**
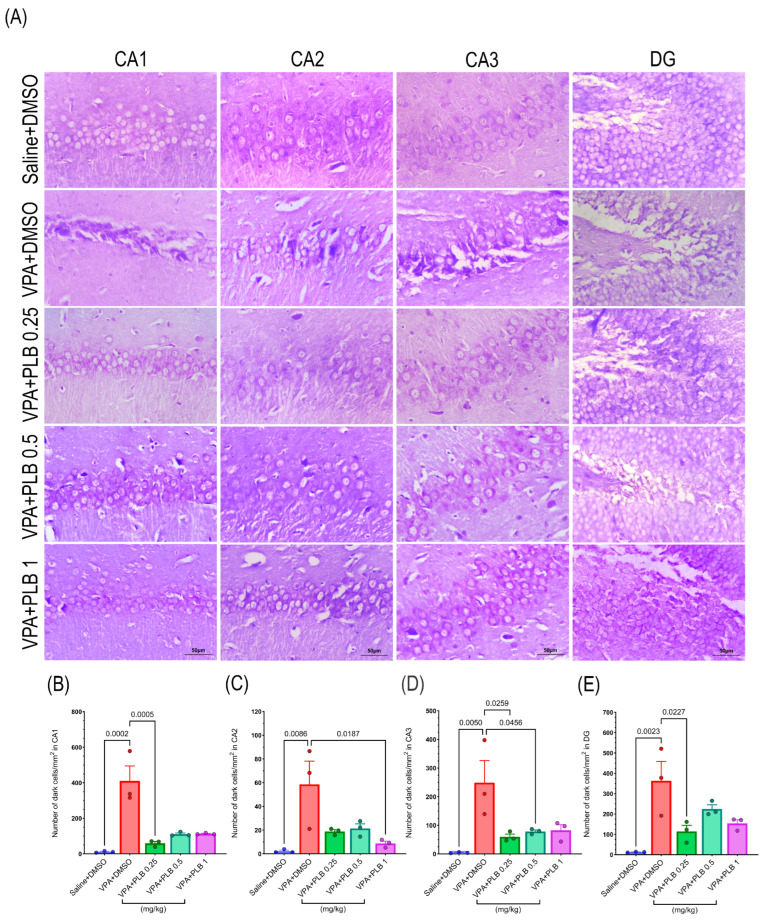
Effect of PLB on the number of hippocampal dark cells in the VPA-induced autism model. (**A**) Nissl staining in the CA1, CA2, CA3, and DG regions of the hippocampus. (**B**–**E**) The quantification results for the number of dark cells. The data are represented as the mean ± SEM. Scale bar: 50 μm, (n = 3).

The Nissl staining data in the hippocampus revealed a significant ANOVA result for the CA1 (X2 = 22.06, df = 4, *p* = 0.0002), CA2 (X2 = 24.06, df = 4, *p* < 0.0001), CA3 (X2 = 20.34, df = 4, *p* = 0.0004), and DG (X2 = 32.83, DF = 4, *p* < 0.0001) regions. The post hoc comparisons showed that the number of dark cells in CA1 was significantly increased in the VPA+DMSO pups (*p* = 0.0006) and VPA+PLB 0.5 pups (*p* = 0.02) compared to the saline+DMSO-treated pups. The number of dark cells in CA1 was significantly decreased in the PLB 0.25-treated pups (*p* = 0.008) compared to the VPA+DMSO pups (Figure 3A,B). In the CA2 region of the hippocampus, the number of dark cells was increased in the VPA+DMSO-treated pups (*p* < 0.0001) compared with the saline+DMSO-treated pups. A reduction in the number of dark cells was found in the VPA+PLB 1 pups (*p* = 0.004) compared to the VPA+DMSO-treated pups (Figure 3A,C). Subsequently, in the CA3 region of the hippocampus, the number of dark cells was significantly decreased in the VPA+DMSO-treated pups (*p* = 0.0008) compared with the saline+DMSO-treated pups. The number of dark cells was significantly decreased in the VPA+PLB 0.25 pups (*p* = 0.02) and VPA+PLB 1 pups (*p* = 0.03) compared with the VPA+DMSO-treated pups (Figure 3A,D). In the DG, there was a significant difference in the number of dark cells in the VPA+DMSO-treated pups (*p* < 0.0001) and VPA+PLB 0.5-treated pups (*p* = 0.001) compared to the saline+DMSO-treated pups. A reduction in the number of dark cells was found in the VPA+PLB 0.25 pups (*p* = 0.001) (Figure 3A,E).

### 3.3. Plumbagin Attenuated the Hippocampal Neuro-Morphological Alterations in the VPA-Induced Autism Model

**Figure 4:**
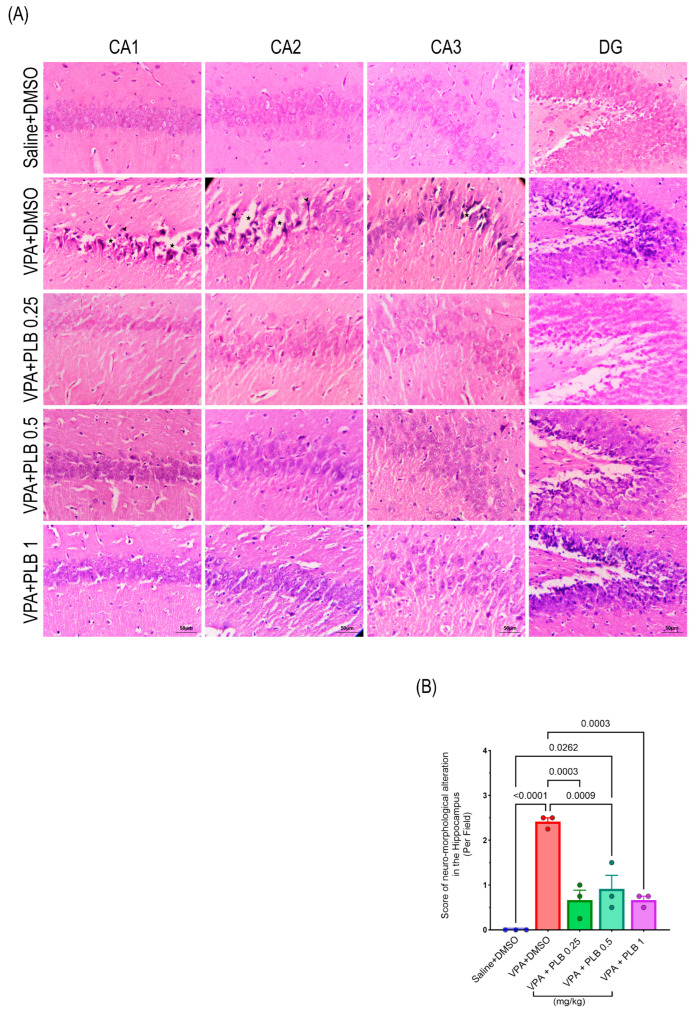
Effect of PLB on neuro-morphological alterations in the VPA-induced autism model. (**A**) Illustrations showing H&E staining in the CA1, CA2, CA3, and DG regions of the hippocampus. (**B**) Neuronal-morphological alterations, such as pyknotic cells with darkly stained nuclei (arrowhead), vacuolation (star), and a disorganized cellular arrangement, were evaluated across the groups. The data are represented as the mean ± SEM. Scale bar: 50 μm, n = 3.

The severity of hippocampal neuro-morphological alterations was assessed by H&E staining (Table 3). In the saline+DMSO-treated pups, round and tightly packed dentate granule cells and pyramidal cells, characterized by light-stained nuclei, were observed; this indicated a normal morphology in the hippocampal regions (CA1, CA2, CA3, and DG). In contrast, pyknotic cells with dark-stained nuclei, vacuolation, and a disorganized cell arrangement were found in the VPA+DMSO-treated pups, indicating severe alterations (Figure 4A). The ANOVA result showed a significant alteration in the neuro-morphology of the hippocampus (x = 26.30, df = 4, *p* < 0.0001). The post hoc multiple comparisons in the VPA+DMSO (*p* < 0.0001) and VPA+PLB 0.5 (*p* = 0.02) pups revealed a significant neuro-morphological alteration compared to the saline+DMSO-treated pups. Additionally, the VPA+PLB 0.25-treated pups (*p* = 0.0003), VPA+PLB 0.5 pups (*p* = 0.0008), and VPA+PLB 1 pups (*p* = 0.0003) exhibited significantly improved neuro-morphological alterations compared to the VPA+DMSO-treated pups (Figure 4B).

### 3.4. Plumbagin Ameliorated the Hippocampal Astrocyte Activation in the VPA-Induced Autism Model

**Figure 5:**
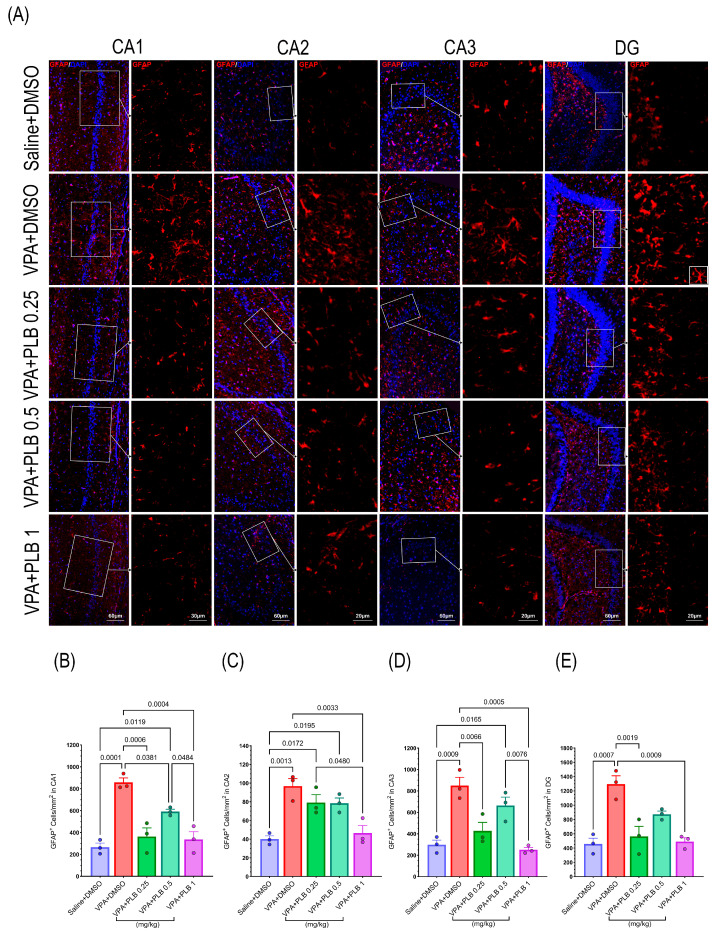
Effect of PLB on hippocampal astrocyte activation in the VPA-induced model. (**A**) Illustration of immunostaining against GFAP as an astrocyte marker in the CA1, CA2, CA3, and DG hippocampus regions. Blue: DAPI: nuclei stain; Red: GFAP: astrocyte marker. (**B**–**E**) The quantification of GFAP immunostaining data. The data are represented as the mean ± SEM. Scale bar: 60 μm and 20 μm, n = 3.

To investigate the level of hippocampal astrocyte activation, we used GFAP immunostaining as an astrocyte marker. The one-way ANOVA showed significant differences between the groups in the CA1 (F (4, 35) = 23.01, *p* < 0.0001), CA2 (F (4, 25) = 12.39, *p* < 0.0001), CA3 (F (4, 25) = 33.33, *p* < 0.0001), and DG (F (4, 25) = 19.79, *p* < 0.0001) regions. Tukey’s post hoc analysis revealed a significant increase in the number of GFAP-positive cells in the CA1 region for both the VPA+DMSO-treated pups (*p* < 0.0001) and the VPA+PLB 0.5-treated pups (*p* = 0.003) compared to the saline+DMSO pups. Conversely, the VPA+PLB 0.25- and VPA+PLB 1-treated pups showed a significant decrease in their GFAP-positive cell levels when compared to VPA-DMSO pups (*p* < 0.0001) (Figure 5A,B).

In the CA2 region, a notable increase in GFAP-positive cells was observed for the VPA+DMSO pups, VPA+PLB 0.25 pups (*p* = 0.02), and VPA+PLB 0.5 pups (*p* = 0.001) compared to the saline+DMSO-treated pups. Additionally, the VPA+PLB 1-treated pups showed a significantly reduced number of GFAP-positive cells compared to the VPA+DMSO-treated pups (*p* = 0.0002) and the VPA+PLB 0.5-treated pups (*p* = 0.008).

In the CA3 region, a significant elevation in the number of GFAP-positive cells was found for the VPA+DMSO-treated pups and the VPA+PLB 0.5-treated pups compared with the saline+DMSO-treated pups (*p* < 0.0001). In contrast, the activity of GFAP-positive cells in the VPA+PLB 0.25-treated pups and VPA+PLB 1-treated pups was significantly decreased compared to the VPA+DMSO and VPA+PLB 0.5 pups (*p* < 0.0001).

In the DG region, a significant increase in GFAP-positive cells was observed for both the VPA+ DMSO (*p* < 0.001) and VPA+PLB 0.5 (*p* = 0.009) groups when compared to the saline+DMSO pups. In parallel, a reduction in GFAP-positive cells was observed for the VPA+PLB 0.25-treated (*p* < 0.0001), VPA+PLB 0.5-treated (*p* = 0.007), and VPA+PLB 1-treated (*p* < 0.0001) pups compared to the VPA+DMSO pups. Furthermore, among the PLB-treated groups, the VPA+PLB 0.25-treated pups exhibited lower GFAP-positive cell counts compared to the VPA+PLB 0.5-treated pups (*p* = 0.01).

### 3.5. Plumbagin Reduced Pro-Inflammatory Cytokine Gene Expression in the Hippocampus

A one-way ANOVA indicated significant differences between the groups for the *IL-1β* (F (4, 15) = 9.79; *p =* 0.0004) and IL-6 (F (4, 15) = 4.07; *p* = 0.01) genes, whereas the differences between groups for *TNF-α* (F (4, 15) = 1.94; *p* = 0.1) were not significant. Tukey’s post hoc test showed a significant elevation in the expression level of *IL-1β* in the VPA+DMSO-treated pups, as well as the VPA+PLB 0.25 (*p* = 0.006) and VPA+PLB 0.5 (*p* = 0.01) groups, compared to the saline+DMSO pups. Additionally, a significant decrease in the expression level of *IL-1β* was observed in the VPA+PLB 1-treated pups compared to the VPA+DMSO pups.

A significant increase in the expression of *IL-6* was detected in the VPA+DMSO group compared to the saline+DMSO-treated pups. Moreover, a reduction in the *IL-6* expression was observed for the VPA+PLB 1-treated pups, but this reduction did not reach statistical significance (*p* = 0.05).

**Figure 6:**
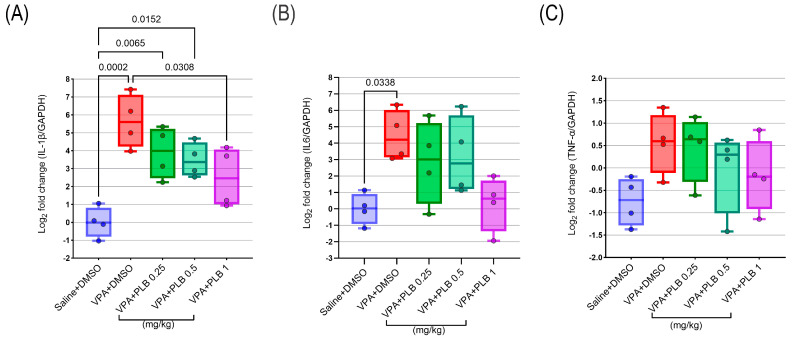
Effect of PLB on the expression levels of *IL-6*, *IL-1β*, and *TNF-α* in the VPA-induced model. (**A**–**C**) Quantification results for the expression levels of *IL-6*, *IL-1β*, and *TNF-α*. The data are represented as the mean ± SEM, n = 4.

In multiple comparisons for the *TNF-α* expression level, no significant changes were observed between any of the groups of treated pups (Figure 6A–C).

## 4. Discussion

In the present study, PLB administration significantly enhanced spatial learning and memory in the VPA-induced model of autism. We demonstrated that PLB reduced astrocyte activation and decreased the levels of pro-inflammatory cytokines, thereby mitigating hippocampal inflammation and ameliorating neuro-morphological alterations.

The hippocampus is an essential part of the CNS and plays a critical role in spatial navigation and memory [27]. Although learning and memory impairment are not core symptoms of autism, they may underlie social behavioral impairments in ASD [28,29]. Dysfunction in the hippocampus is a key factor contributing to impairments in social–cognitive memory processing in individuals with ASD and the animal model of autism [2,29]. Our findings confirm that the administration of VPA during gestational day 12.5 is associated with cognitive deficits, as evidenced by a longer escape latency and traveled distance, along with a decreased percentage of time spent in the target quadrant. Although hyperactivity and anxiety were not investigated, the increased velocity and traveled distance by the VPA-exposed groups compared to saline exposure may reflect these behavioral changes related to ASD [30,31]. However, this interpretation remains controversial, as some studies have shown that the cognitive function in the ASD model may stay intact or even enhance, depending on factors such as the age, sex, and experimental design [32,33]. Notably, in the present study, treatment with PLB improved the cognitive performance, as evidenced by enhancements in spatial learning and preference memory. In this regard, decreasing the velocity in the PLB 1 mg/kg group is probably associated with a decline in motivation to find the platform due to decreasing hyperactivity and anxiety. In our previous study, we found that PLB improved social behaviors in the VPA model of autism [20]. Hence, we suggested that an improving cognitive function could underlie an enhanced social behavioral performance. Additionally, a previous report showed that PLB enhanced the memory in Alzheimer’s disease (AD) and amnesia models in rodents [16,17].

The exact pathophysiological mechanisms underlying cognitive deficits in ASD is not completely clear; however, numerous examples of clinical evidence and animal models have confirmed the neuroanatomical and pathophysiological abnormalities of the hippocampus in individuals with ASD [29,34,35]. A histopathological assessment aimed at identifying the cause of the cognitive deficits revealed that prenatal VPA administration enhanced neural cell loss and induced neuronal–morphological alterations in the hippocampal regions (CA1, CA2, CA3, and DG). The hippocampus showed a disorganized cellular structure, reflected by the reduced compactness of pyramidal and granular layers and diffuse boundaries. This evidence was in line with previous histological studies of postmortem autistic patients and supports the validity of the VPA model in rodents [7,36,37]. The results of our findings revealed that PLB at 0.25 mg/kg or 1 mg/kg reduced neural cell loss and alleviated neuronal–morphological alterations in pups administered VPA. These results are in agreement with a previous study showing that the neuroprotective effects of PLB are associated with enhancing the PI3K/Akt and ERK signaling pathways [38]. Furthermore, PLB may suppress neuronal death and apoptosis by enhancing Nrf2/ARE expression and regulating the BDNF signaling pathway [39].

Astrocytes have an essential role in synapse formation and function, regulating neurotransmitter homeostasis and modulating synaptic connections [40]. Moreover, astrocytes contribute during neurodevelopment in neurogenesis and neuronal plasticity [41]. During immune responses, reactive astrocytes act as a double-edged sword. On one hand, astrocytes act as neuroprotective glial cells that upregulate several neuroprotective factors, promoting the synaptic repair, growth, and survival of neurons. On the other hand, during the acute stage, reactive astrocytes exert neurotoxicity by releasing more chronic pro-inflammatory cytokines and leading to the loss of synaptogenesis and neuronal death [41,42]. The inflammatory response and astrogliosis play essential roles in histological damage and are likely considered one of the main factors in the pathogenesis of ASD; therefore, improving astrocyte function may serve as an effective therapeutic strategy for individuals with autism [43]. Our findings indicated an enhancement of activated astrocytes in the hippocampal regions of the VPA-administered group during adolescence, which is in agreement with previous studies on a VPA model of autism [25,44]. In the present study, treatment with PLB at 0.25 mg/kg or 1 mg/kg reduced astrocyte activation in the CA1, CA3, and DG regions; however, only PLB at 1 mg/kg demonstrated efficacy in the CA2 region of the hippocampus. This phenomenon may be attributed to the unique morphological and physiological characteristics of CA2, which distinguish it from CA1, CA3, and DG [45]. In this regard, our previous cohort study demonstrated that PLB reduced glial activation and oxidative stress in the cerebellum, which may be associated with improvements in behavioral deficits related to ASD [20].

Alterations in immune cell function promote neuroinflammation in the brain, which may affect cognitive functions [38,41]. Since cytokine alterations are a potential biomarker in diagnosing ASD, recent studies have reported increased levels of IL-6, IL-1β, and TNF-α in the blood, cerebrospinal fluid (CSF), and brain of ASD patients as well as in the VPA rodent model [37,42]. Our findings indicated a significant upregulation of the hippocampal *IL-6* and *IL-1β* expression levels in pups administered VPA. However, elevated levels of TNF-α were not observed in rats who received VPA, possibly due to limitations in the sample size, the age, and biological variability [43].

The administration of PLB at 1 mg/kg mitigated the upregulation of inflammatory cytokines in the hippocampus as well as astrocyte activation, implying a strong anti-inflammatory effect in the hippocampus. Some evidence indicates a relation between reduced levels of pro-inflammatory cytokines and improved autistic-like behaviors [44,45]. Additionally, the anti-inflammatory response of PLB has been evaluated in rats with chronic periodontitis. It was shown that PLB significantly decreased the levels of pro-inflammatory cytokines via the MAPK, NF-B, and JAK/STAT signaling pathways [46]. In one study, PLB was found to suppress neuroinflammation by modulating the NF-κB and Nrf-2 signaling pathways in an experimental model of AD [46]. PLB also mitigates the expression levels of cytokines in myocardial ischemia by regulating reactive oxygen species [47].

## 5. Limitations and Future Directions

While the results are promising, the limitations warrant careful interpretation and further research. First, the low bioavailability of PLB could have limited its effectiveness. Different synthetic analogues and nano-formulations of PLB are needed to enhance the bioavailability of PLB and improve its pharmacokinetic characteristics. Although species-specific data are limited, differences in metabolism and absorption between animal models may affect PLB efficacy and should be considered in future studies. Second, hippocampal neuro-morphological alterations were assessed via H&E staining in a blinded evaluation. To further improve the objectivity of the histological data, future studies should include formal inter-rater reliability metrics (e.g., Cohen’s kappa) and apoptosis markers (e.g., caspase-3 and TUNEL). Third, the lack of the direct quantification of neuronal cell death limited our ability to draw definitive conclusions regarding the relationship between neuronal loss and the Nrf2/ARE and BDNF pathways in ASD. Future studies should incorporate an examination of cell death markers to elucidate PLB’s neuroprotective potential. Fourth, there is compelling evidence on astrocyte activation in ASD; further research is needed to evaluate other astrocyte markers, such as AQP4, connexin30, and connexin43, in the hippocampus, which play an important role in synaptic plasticity, cognition, and learning. Notably, conventional immunofluorescence imaging’s limited spatial resolution may hinder accurate GFAP quantification, especially in fine astrocyte processes. This limitation highlights the importance of using more molecular markers alongside GFAP assessments to fully understand the astrocyte function in ASD. Fifth, the interpretation of the cytokine expression data should be approached with caution. Several factors, such as the limited sample size and potential age-related variability, may have influenced the cytokine patterns. Future studies with a larger sample size and age-matched controls are needed to validate these findings.

## 6. Conclusions

VPA exposure during pregnancy exhibited cognitive deficits along with morphological alterations and neuroinflammation in the hippocampus. Additionally, PLB, a natural compound with multi-functional effects, ameliorated several cellular and molecular defects in the hippocampus. It appears that PLB, particularly at a dose of 1 mg/kg, mediated its beneficial impacts by attenuating inflammation and enhancing neuroprotection through reducing dark cells and improving the neuro-morphological alterations within the hippocampus. Moreover, alongside our recent findings in the cerebellum, these results suggested that PLB, through its balanced modulation of anti-inflammatory, anti-oxidative, and neuromodulatory pathways, could be a promising candidate for ASD intervention, targeting both behavioral impairments and neuroinflammatory mechanisms across critical brain areas. Despite certain limitations, the current findings suggest a valuable foundation for further studies aimed at elucidating the exact mechanisms and signaling pathways through which PLB exerts its beneficial effects in autism, extending beyond adolescence into adulthood.

## Figures and Tables

**Table 1:** The count of pyknotic cells with dark nuclei.

Score	CA1/CA3	CA2	DG
None (0)	0–5	0–1	0–10
Moderate (1)	5–15	1–5	10–25
Mild (2)	15–30	5–10	25–50
Severe (3)	30>	10>	50>

**Table 2:** The primer sequences.

Gene	5′ Forward 3′	3′ Reverse 5′
*GAPDH*	GACGGCCGCATCTTCTTGTAG	GACATACTCAGCACCAGCATCACC
*IL-6*	TGATGGATGCTTCCAAACTG	GAGCATTGGAAGTTGGGGTA
*IL-1β*	GCTGTGGCAGCTACCTATGTCTTG	AGGTCGTCATCATCCCAC GAG
*TNF-α*	AAATGGGCTCCCTCTCATCAGTTC	TCTGCTTGGTGGTTTGCTACGAC

**Table 3:** Scoring of the neuro-morphological alteration in the hippocampus.

Lesions/Regions	Saline+DMSO	VPA+DMSO	VPA+PLB 0.25 (mg/kg)	VPA+PLB 0.5 (mg/kg)	VPA+PLB 1 (mg/kg)
**CA1**					
Dark neurons	−	+++	+	+	+
Vacuolation	−	++	+	−	+
Disorganized cell arrangement	−	++	−	−	−
**CA2**					
Dark neurons	−	+++	+	+	+
Vacuolation	−	+++	−	++	+
Disorganized cell arrangement	−	+++	−	−	−
**CA3**					
Dark neurons	−	+++	−	+	+
Vacuolation	−	++	+	−	+
Disorganized cell arrangement	−	+	−	−	−
**DG**					
Dark neurons	−	+++	++	+++	+
Vacuolation	−	++	++	++	+
Disorganized cell arrangement	−	++	−	+	−

Pyknotic cells with dark nuclei: −: None, +: Moderate, ++: Mild, +++: Severe.

## Data Availability

The raw data supporting the conclusions of this article will be made available by the authors upon reasonable request. The data are not publicly available due to specific ethical and privacy considerations.

## Abbreviations

Autism spectrum disorder	ASD
Plumbagin	PLB
Valproic acid	VPA
Morris water maze	MWM
Quantitative real-time polymerase chain reaction	qRT-PCR
Hematoxylin–eosin staining	H&E
Interleukin 6	IL-6
Blood–brain barrier	BBB
Nuclear factor erythroid 2-related factor 2	Nrf2
Nuclear factor κB	NF-κB
Dimethylsulfoxide	DMSO
Embryonic day	E
Postnatal day	PND
North	N
South	S
East	E
West	W
Human visual system	HVS
Paraformaldehyde	PFA
Phosphate-buffered saline solution	PBS
Cornu ammonis	CA
Dentate gyrus	DG
Glial fibrillary acetic acid protein	GFAP
Normal goat serum	NGS
Immunoglobulin	IgG
4′,6-diamidino-2-phenylindole	DAPI
Ribonucleic acid	RNA
Complementary deoxyribonucleic acid	cDNA
Glyceraldehyde-3-phosphate dehydrogenase	GAPDH
Brain-derived neurotrophic factor	BDNF
Janus kinase/signal transducers and activators of transcription	JAK/STAT
Alzheimer’s disease	AD
